# The impact of eliminating age inequalities in stage at diagnosis on breast cancer survival for older women

**DOI:** 10.1038/bjc.2015.51

**Published:** 2015-03-03

**Authors:** M J Rutherford, G A Abel, D C Greenberg, P C Lambert, G Lyratzopoulos

**Affiliations:** 1 Department of Health Sciences, University of Leicester, Leicester LE1 7RH UK; 2 Cambridge Centre for Health Services Research, Department of Public Health and Primary Care, University of Cambridge, Cambridge CB2 0SR, UK; 3 National Cancer Registration Service, Public Health England, Eastern Office, Cambridge CB22 3AD, UK; 4 Department of Medical Epidemiology and Biostatistics, Karolinska Institutet, Stocholm SE-171 77, Sweden; 5 Health Behaviour Research Centre, Department of Epidemiology and Public Health, University College London, UCL, 1-19 Torrington Place, London WC1E 6BT, UK

**Keywords:** avoidable deaths, age inequalities, excess mortality models

## Abstract

**Background:**

Older women with breast cancer have poorer relative survival outcomes, but whether achieving earlier stage at diagnosis would translate to substantial reductions in mortality is uncertain.

**Methods:**

We analysed data on East of England women with breast cancer (2006–2010) aged 70+ years. We estimated survival for different stage-deprivation-age group strata using both the observed and a hypothetical stage distribution (assuming that all women aged 75+ years acquired the stage distribution of those aged 70–74 years). We subsequently estimated deaths that could be postponed beyond 5 years from diagnosis if women aged 75+ years had the hypothetical stage distribution. We projected findings to the English population using appropriate age and socioeconomic group weights.

**Results:**

For a typically sized annual cohort in the East of England, 27 deaths in women with breast cancer aged 75+ years can be postponed within 5 years from diagnosis if their stage distribution matched that of the women aged 70–74 years (4.8% of all 566 deaths within 5 years post diagnosis in this population). Under assumptions, we estimate that the respective number for England would be 280 deaths (5.0% of all deaths within 5 years post diagnosis in this population).

**Conclusions:**

The findings support ongoing development of targeted campaigns aimed at encouraging prompt presentation in older women.

Across Europe, older patients have benefited least from the substantial improvements in cancer survival in recent decades (Quaglia *et al*, 2009). Age gaps in cancer survival have been greatest in the UK and Ireland (De Angelis *et al*, 2014). Although some of these age inequalities may reflect sub-optimal management (Lavelle *et al*, 2007), another part may reflect that older patients are diagnosed at more advanced disease stage. For example, in England, older persons are at a greater risk of advanced stage at diagnosis of melanoma, endometrial and breast cancers (Lyratzopoulos *et al*, 2013a).

In England, about 6000 deaths in women aged 70 years or over are attributed to breast cancer each year (Office for National Statistics, 2013), and around a third of all new diagnoses of breast cancer occur in this age group (Cancer Research UK (CRUK) Website, 2014). In theory, decreasing the proportion of older women who are diagnosed with advanced-stage breast cancer should help prolong survival. Establishing the size of anticipated reduction in mortality using empirical evidence from population-based cohorts would be useful. Against this background, we have set out to examine the potential for preventing deaths within 5 years from diagnosis of breast cancer in older women that could result from eliminating age inequalities in stage at diagnosis.

## Materials and methods

### Data

We analysed time from diagnosis to death for East of England women aged 70 years or over with a new diagnosis of breast cancer (International Classification of Diseases–10 site code C50) during 2006–2010, with follow-up on mortality until 15 March 2012. As described previously (Rutherford *et al*, 2013b), the data were extracted from the (former) Eastern Cancer Registration and Information Centre (ECRIC), a cancer registry covering a population of ∼5.7 million across the East of England region. Stage at diagnosis was assigned by medical practitioners with specialist expertise, based on clinical, imaging and pathological information according to the TNM classification (Sobin and Fleming, 1997). Socioeconomic status groups (one least deprived, five most deprived) were defined ecologically, using national quintiles of the income domain of the Index of Multiple Deprivation 2010 score of the Lower Super Output Area of patients' residence at diagnosis (Department for Communities and Local Government, 2011). We categorised age into four groups: 70–74, 75–79, 80–84 and 85+ years. Deprivation and age information were available for all patients. The data on age group and deprivation are tabulated in Table 1.

### Analysis

We used a similar approach to Rutherford *et al* (2013b) but with matching of stage distributions across age rather than deprivation groups. Full details of the method are described in the methods section and appendix of the previously published paper (Rutherford *et al*, 2013b). Briefly, we fitted a flexible parametric excess mortality model (Nelson *et al*, 2007; Royston and Lambert, 2011) to estimate the effect of age, stage and deprivation status on excess mortality. We then calculated the proportion of women diagnosed at each stage for all deprivation-age group strata to obtain the relevant weights for the stage standardisation. Using the model estimates and these weights, we calculated stage-standardised survival for each age and deprivation group using two different stage distributions; one based on the observed stage distribution in each category and the second based on the stage distribution (in the respective deprivation group) for women aged 70–74 years. We calculated the difference between these two standardised survival estimates while accounting for other cause mortality, and we report the number of deaths postponed beyond given points during the follow-up period (i.e., beyond 5 years) by ‘equalising' the stage distribution of each age group to match that of women in the 70- to 74-year group. We used a complete case analysis approach and deprivation quintile-specific life-tables for all survival estimates (Cancer Research UK Cancer Survival Group, 2006). Confidence intervals for the number of postponable deaths were calculated using the delta method using a similar approach to that described by Seppa *et al* (2012).

We also approximated the number of deaths that could be postponed in the whole of England rather than just the East of England region by appropriately weighting the estimates (Rutherford *et al*, 2013b) to match the age and socioeconomic group distribution for those aged 70+ years in England as a whole. This involved re-weighting the estimates to account for sample size and compositional differences (e.g., regarding deprivation group) between the populations of East of England and England.

### Supplementary analysis

We examined the potential for confounding by tumour type by exploring associations between age group and morphology, and morphology and stage. Using International Classification of Diseases-Oncology morphology codes, four tumour groups (infiltrating ductal carcinoma, infiltrating lobular carcinoma, mixed infiltrating ductal and lobular carcinoma, and other unspecified types) were used for this analysis.

## Results

There were 6478 women aged ⩾70 years with complete information on stage at diagnosis included in subsequent analysis (88% of an initial total of 7331 women). The proportion of women with missing stage information was 6.2%, 7.6%, 12.8% and 20% for age groups 70–74 years, 75–79 years, 80–84 years and 85+ years, respectively. As previously reported (Lyratzopoulos *et al*, 2013a), among women with known stage, the proportion diagnosed in earlier stage progressively decreases with age over 70 years. For example, the proportion of women diagnosed at stage I is 39% for women aged 70–74 years, whereas it is only 23% for the 85+ age group; these age gradients are similar across deprivation groups (Table 1).

**Figure 1:**
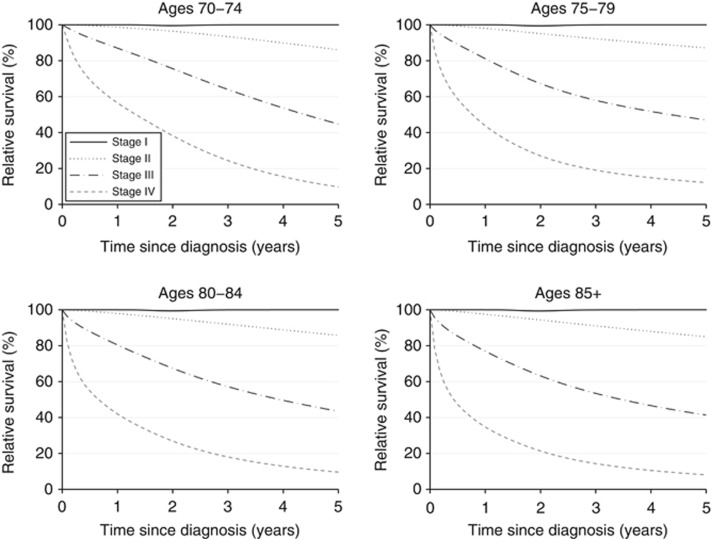
Stage-specific survival across the age groups 70–74, 75–79, 80–84 and 85+ years.

Stage-specific survival is markedly different for stage I compared with stages III or IV, across age groups (Figure 1). Therefore, if the distribution of stage at diagnosis for the older women matched that of those aged 70–74 years, there should be measurable improvements in survival, translating to a number of all-cause deaths that could be postponed.

Figure 1 shows the stage-specific survival for women aged 70–74, 75–79, 80–84 and 85+ years. Relative survival for women diagnosed at stage I is nearly 100% during the entire follow-up period (i.e., up to 5 years from diagnosis) and for all age groups. In other words, women diagnosed at stage I experience almost no excess mortality compared with those in the general population without breast cancer. Stage-specific survival is fairly similar across all age groups (Figure 1). The relative survival for patients diagnosed at stage III or IV is quite poor across all age groups (for example, the 5-year relative survival for women diagnosed at stage III is ∼40%, Figure 1), indicating that these women have a considerable excess mortality owing to breast cancer.

**Figure 2:**
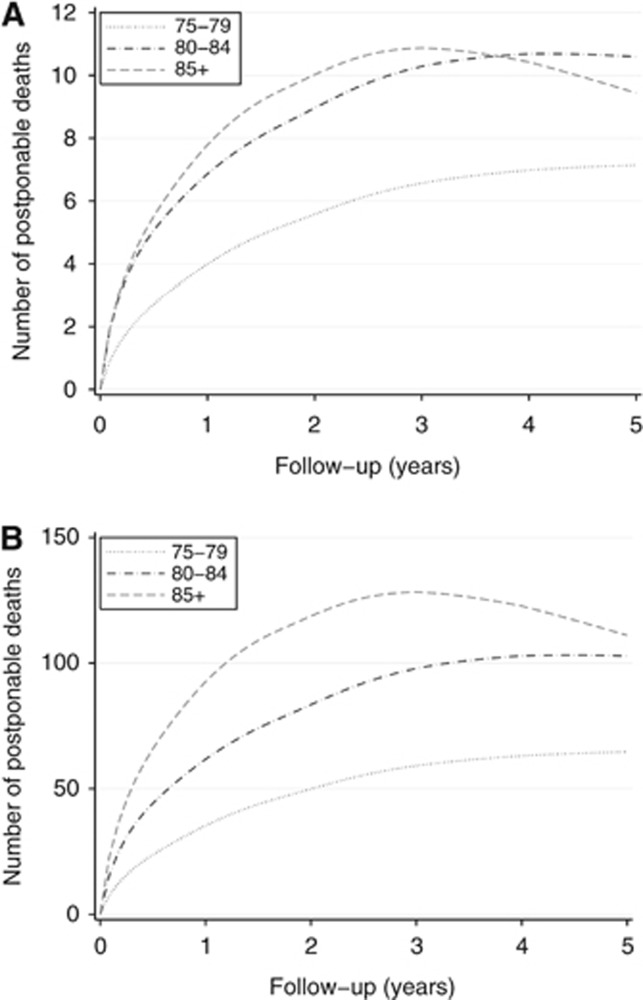
**Number of avoidable deaths within 5 years from diagnosis of cancer.** (**A**) East of England. (**B**) England. Note the different *y*-axis scales in the two sub-figures. Total values across all age groups at 5 years: 27.17 deaths in East of England, 278.59 deaths for England.

Figure 2 shows the number of deaths that can be postponed beyond certain points during the follow-up period by achieving earlier stage distribution in the three older age groups (i.e., by matching their stage distribution to that of women aged 70–74 years within the same deprivation groups). Figure 2A shows the relevant estimates for the East of England for (a typical annual cohort of 1296 cases) showing that 27 deaths (point estimate of 27.2 with 95% CI 25.2 to 29.2) can be postponed to a time point beyond 5 years from diagnosis. This would represent 4.8% of all 566 deaths calculated to occur within 5 years from diagnosis in women with breast cancer aged >70 years in East of England. Figure 2B shows the re-weighted estimates for England using 2009 incidence figures for those aged ⩾70 years—about 280 deaths postponed beyond 5 years among older women, or 5.0% of all deaths in the respective population.

## Discussion

There is potential for reducing excess mortality from breast cancer by eliminating age inequalities in stage at diagnosis in women aged 70 years or over. This reduction in excess mortality will have a direct impact on all-cause survival estimates, and we estimate that around 280 deaths would be postponed beyond 5 years for a typical annual cohort size of older (70+) women with breast cancer in England.

Strengths of our study include the use of a large population-based sample with good quality and completeness of information on stage at diagnosis, and the use of a flexible parametric model, allowing for the smooth estimation of excess mortality throughout the follow-up period while appropriately accounting for the effects of deprivation and age (Rutherford *et al*, 2013a). The methodology that we have used in this paper can help support monitoring of the impact of population-based early awareness and detection interventions (Be Clear On Cancer—Breast Cancer in Over 70s Campaign, 2014).

We restricted our study population to a historical cohort of East of England women (2006–10) with both high level of completeness of information on stage at diagnosis and an adequate follow-up period. As recording of stage information becomes increasingly complete and consistent across England, future work should aim to encompass more recent cohorts of patients using nationwide data. Limiting to regional data means that we have small numbers for some of the cells in Table 1, particularly for the most-deprived patients and late-stage disease. Extrapolating the regional estimates to England as a whole makes the assumption that patients with breast cancer in England do not differ from those in the East of England in terms of expected survival, the effect of the covariates on relative survival and the distribution of stage at diagnosis. Overall, these assumptions appear to be fairly reasonable, particularly given only modest variation in short-term relative survival for breast cancer patients between the English regions (Rachet *et al*, 2009).

To further aid interpretation and to contextualise the findings, we would like to draw attention to the fact that all deaths estimated in our paper are actually postponed (an alternative term used for this measure is avoidable deaths)—the entire cohort will eventually diminish to 0 if follow-up is extended long enough. For women aged 85+ years in particular, the estimated number of postponed deaths begins to decrease within 5 years from diagnosis (Figure 2), as other cause mortality is particularly high in that age group. Further, the findings need to be interpreted in the context of previous work examining potential survival gains by eliminating socioeconomic inequalities in stage at diagnosis of breast cancer among women of any age (Rutherford *et al*, 2013b). In contrast, the present study solely focuses on age inequalities in stage at diagnosis among older women. Combined elimination of inequalities in stage at diagnosis both by socioeconomic and older age groups would be associated with a greater number of deaths that can be postponed beyond 5 years from diagnosis in the population.

Three issues deserve further discussion. First, our method does not make any adjustment for differences in tumour type across age groups. We have shown in supplementary analyses that differences in morphology by age are unlikely to be large (Supplementary Online Material Tables 1 and 2). Although there are some differences in the stage distribution across morphology groups (Supplementary Online Material Table 3), tumour type does not have a large impact on survival in addition to stage. Although breast cancer is known to be a heterogeneous disease, most of the tumour type differences relate to pre-menopausal compared with post-menopausal women (Anderson *et al*, 2014). In contrast, our study population only included (post-menopausal) women aged ⩾70 years.

Second, we excluded women below the age of 70 years owing to the impact of screening. Screen detection has an impact on stage at diagnosis (the great majority of screen-detected cases will be diagnosed at stages I or II) and could introduce differential lead time bias in the survival estimates. Evidence does not, at least currently, support the offer of screening in older women (Independent UK Panel on Breast Cancer Screening, 2012; de Glas *et al*, 2014), although this is the subject of ongoing research (CRUK Website—A study to evaluate an age extension to the NHS Breast Screening Programme, 2014). However, our method could be sensitive to the use of screening in our sample. The proportion of women in our study population who were screening-detected differs by age (26%, 12%, 3% and 1% for women aged 70–74, 75–79, 80–84 and 85+ years, respectively). It is likely that these differences largely reflect age variation in use (as opposed to effectiveness) of screening. Owing to the level of screening use in women aged 70–74, our global estimate of avoidable all-cause deaths in women aged 70 years or older may be upwardly inflated. Nevertheless, further potential gains in the number of postponable deaths in women older than 70 could be made by achieving a (more favourable) stage distribution that would be seen for patients aged 65–69 years. However, this comparison is made difficult by the high use of screening in this group (over half of all women aged 65–69 are detected by screening).

Finally, older age is associated with both a greater risk of advanced stage at diagnosis and missing stage information. Therefore, and as our findings are based on a complete case analysis, the true potential for avoidable deaths may have been under-estimated. In addition, the higher concentration of (excluded) cases with missing stage in older age groups leads to differential reduction of the sample size, again resulting in underestimation of the true potential for reducing deaths in older women. On the other hand, women with missing stage disease also have fairly poor survival, and therefore our stage-specific survival estimates may be upwardly biased to a small degree.

In their largest part, age differentials in stage at diagnosis of breast cancer in women aged ⩾70 years are likely to reflect age differences in promptness of presentation (i.e., age differences in the patient interval) as opposed to differences in diagnostic intervals after presentation. This is because after seeking medical help with relevant symptoms, the great majority of English women subsequently diagnosed with breast cancer are referred very promptly independently of their age (median primary care interval=0 days, interquartile range: 0–1; Lyratzopoulos *et al*, 2013b), with concordant evidence from Scotland (Baughan *et al*, 2009). Further, older age confers no disadvantage in speed of specialist referral (Lyratzopoulos *et al*, 2012).

We did not have information on co-morbidity in our study, which increases with age. However, as we estimate survival within age groups, this will lessen the impact of differential co-morbidity by age. Further, evidence from the US indicates that greater levels of co-morbidity may be associated with earlier stage at diagnosis of breast and colorectal cancer, possibly because of greater opportunities to detect cancer early in more co-morbid patients through more frequent care appointments (Keating *et al*, 2005, Zafar *et al*, 2008). Therefore, the fact that older women could be expected to have greater levels of co-morbidity could facilitate rather than impede earlier stage at diagnosis.

Our findings therefore provide support both for further research to establish the causes of age inequalities in stage at diagnosis of breast cancer and ongoing public health campaigns aimed to encourage prompt presentation in older women (Be Clear On Cancer—Breast Cancer in Over 70s Campaign, 2014). Such campaigns can, for example, aim to increase awareness of cancer signs and symptoms (a conceptual surrogate of the patient interval; Lyratzopoulos, 2014), which is lower among older patients (Robb *et al*, 2009; Forbes *et al*, 2011), or they can aim to increase the public understanding of the age-dependent nature of breast cancer risk, which is low in the general English population (Forbes *et al*, 2013). Subject to appropriate development, validation and evaluation, campaigns that target women aged 70+ years should be encouraged, as they can lead to substantial reductions in avoidable mortality through earlier stage diagnosis of breast cancer in these women.

## Figures and Tables

**Table 1:** Stage distributions across age and deprivation groups

	**Stage I**	**Stage II**	**Stage III**	**Stage IV**	**Total**
**Most affluent**					
70–74	182 (41.27)	200 (45.4)	40 (9.1)	19 (4.3)	441 (100)
75–79	173 (37.0)	220 (47.0)	55 (11.8)	20 (4.3)	468 (100)
80–84	103 (32.2)	155 (48.4)	38 (11.9)	24 (7.5)	320 (100)
85+	70 (22.7)	168 (54.6)	47 (15.3)	23 (7.5)	308 (100)
**Deprivation group 2**					
70–74	183 (39.1)	220 (47.0)	55 (11.8)	20 (4.3)	468 (100)
75–79	145 (30.8)	133 (44.9)	31 (10.5)	21 (7.1)	471 (100)
80–84	93 (26.7)	163 (46.8)	50 (14.4)	42 (12.1)	348 (100)
85+	91 (24.1)	185 (48.9)	67 (17.7)	35 (9.3)	378 (100)
**Deprivation group 3**					
70–74	174 (39.0)	193 (43.3)	38 (8.5)	41 (9.2)	446 (100)
75–79	158 (35.5)	197 (44.3)	49 (11.0)	41 (9.2)	445 (100)
80–84	118 (29.4)	190 (47.3)	47 (11.7)	47 (11.7)	402 (100)
85+	94 (23.6)	199 (50.0)	68 (17.1)	37 (9.3)	398 (100)
**Deprivation group 4**					
70–74	111 (37.5)	133 (44.9)	31 (10.5)	21 (7.1)	296 (100)
75–79	91 (27.7)	156 (47.6)	36 (11.0)	45 (13.7)	328 (100)
80–84	69 (24.0)	149 (51.9)	41 (14.3)	28 (9.8)	287 (100)
85+	71 (22.9)	158 (51.0)	63 (20.3)	18 (5.8)	310 (100)
**Most deprived**					
70–74	41 (40.6)	43 (42.6)	9 (8.9)	8 (7.9)	101 (100)
75–79	32 (33.3)	45 (46.9)	12 (12.5)	7 (7.29)	96 (100)
80–84	19 (25.3)	37 (49.3)	16 (21.33)	3 (4.0)	75 (100)
85+	19 (20.7)	39 (42.4)	26 (28.3)	8 (8.7)	92 (100)
**Total (over deprivation groups)**					
70–74	691 (39.4)	769 (43.9)	172 (9.8)	120 (6.9)	1752 (100)
75–79	599 (33.1)	856 (47.4)	203 (11.2)	150 (8.3)	1808 (100)
80–84	402 (28.1)	694 (48.5)	192 (13.4)	144 (10.1)	1432 (100)
85+	345 (23.22)	749 (50.4)	271 (18.2)	121 (8.1)	1486 (100)
**Total (overall)**					
70+	2037 (31.4)	3068 (47.4)	838 (12.9)	535 (8.26)	6478 (100)
