# Supplementary material for: The impact of eliminating age inequalities in stage at diagnosis on breast cancer survival for older women
Source: Br J Cancer. 2015 Mar 3;112(Suppl 1):S124–8. doi: 10.1038/bjc.2015.51 (PMC4385985; doi:10.1038/bjc.2015.51)
Supplement: Supplementary Table 1 [file bjc201551x1.docx]

| **Tumour Type** | **70-74** | **75-79** | **80-84** | **85+** | **Total** |
| --- | --- | --- | --- | --- | --- |
| Infiltrating ductal carcinoma | 1,259 (71.86) | 1,236 (68.36) | 919 (64.18) | 898 (60.43) | 4,312 (66.56) |
| Infiltrating lobular carcinoma | 246 (14.04) | 237 (13.11) | 204 (14.25) | 184 (12.38) | 871 (13.45) |
| Mixed infiltrating ductal and lobular carcinoma | 106 (6.05) | 106 (5.86) | 69 (4.82) | 55 (3.70) | 336 (5.19) |
| Other and  unspecified | 141 (8.05) | 229 (12.67) | 240 (16.76) | 349 (23.49) | 959 (14.81) |
