# Supplementary material for: The impact of eliminating age inequalities in stage at diagnosis on breast cancer survival for older women
Source: Br J Cancer. 2015 Mar 3;112(Suppl 1):S124–8. doi: 10.1038/bjc.2015.51 (PMC4385985; doi:10.1038/bjc.2015.51)
Supplement: Supplementary Table 2 [file bjc201551x2.docx]

| **Tumour Type** | **70-74** | **75-79** | **80-84** | **85+** | **Total** |
| --- | --- | --- | --- | --- | --- |
| Infiltrating ductal carcinoma | 78.15% | 78.28% | 77.10% | 78.98% | 78.13% |
| Infiltrating lobular carcinoma | 15.27% | 15.01% | 17.11% | 16.19% | 15.78% |
| Mixed infiltrating ductal and lobular carcinoma | 6.58% | 6.71% | 5.79% | 4.84% | 6.09% |
