# Supplementary material for: The impact of eliminating age inequalities in stage at diagnosis on breast cancer survival for older women
Source: Br J Cancer. 2015 Mar 3;112(Suppl 1):S124–8. doi: 10.1038/bjc.2015.51 (PMC4385985; doi:10.1038/bjc.2015.51)
Supplement: Supplementary Table 3 [file bjc201551x3.docx]

| **Tumour Type** | **Stage I** | **Stage II** | **Stage III** | **Stage IV** | **Total** |
| --- | --- | --- | --- | --- | --- |
| Infiltrating ductal carcinoma | 1,453 (33.70) | 2,064 (47.87) | 518 (12.01) | 277 (6.42) | 4,312 (100) |
| Infiltrating lobular carcinoma | 231 (26.52) | 428 (49.14) | 130 (14.93) | 82 (9.41) | 871 (100) |
| Mixed infiltrating ductal and lobular carcinoma | 125 (37.20) | 159 (47.32) | 38 (11.31) | 14 (4.17) | 336 (100) |
| Other and  unspecified | 228 (23.77) | 417 (43.48) | 152 (15.85) | 162 (16.89) | 959 (100) |
| **Total** | 2,037 (31.44) | 3,068 (47.36) | 838 (12.94) | 535 (8.26) | 6,478 (100) |
